# Strategies for effective and efficient delivery of primary health care by village health workers: A scoping review using the Rodgers’ Evolutionary Concept Analysis Framework

**DOI:** 10.4102/phcfm.v16i1.4554

**Published:** 2024-12-18

**Authors:** Ofhani Munyai, Azwinndini G. Mudau, Ntsieni S. Mashau

**Affiliations:** 1 Department of Public Health, Faculty of Health Sciences, University of Venda, Thohoyandou, South Africa

**Keywords:** antecedents, attributes, consequences, PHC, VHWs, strategy development

## Abstract

**Background:**

Village health workers (VHWs), popularly known as community health workers (CHWs) in some contexts and settings, should effectively complement health care providers in primary health care (PHC) delivery in Zimbabwe. However, they continue to offer services that do not address current and emerging health issues.

**Aim:**

This study aims to review the literature and develop a conceptual framework to improve the effectiveness and efficiency of VHWs in service delivery.

**Method:**

Rodgers’ evolutionary framework was used to analyse the concept of ‘strategies for effective and efficient delivery of PHC by VHWs’. Articles and reports published in English from 2010 to 2022 in peer-reviewed journals from the PUBMED, EBSCO, ScienceDirect and Google Scholar databases were reviewed.

**Results:**

After screening and removal of duplicates a total of 52 articles and two reports were reviewed to identify antecedents, attributes and consequences of strategies to improve PHC services by village health workers. The antecedents included an enabling work environment, community participation, motivation, incentives, integration of community health into national health systems, and information and communication technology. The attributes consisted of ongoing training and skills development, mutual respect and trust, enhanced contact between VHWs and communities and supportive supervision. The consequences were equitable access to and improved quality of PHC service delivery.

**Conclusion:**

The concept of PHC service delivery by VHWs has evolved from health promotion to curative care through task shifting and is now an integral part of the health system. A supportive and enabling work environment, anchored in community participation, empowers VHWs to deliver equitable services effectively and efficiently.

**Contribution:**

The development of strategies for improving VHW service delivery and a conceptual framework informed by findings from the reviewed literature.

## Introduction

The conference of Alma Ata in 1978 provided a radical declaration towards the achievement of universal health coverage (UHC) through primary health care (PHC) services which are community-driven, equitable and quality health care.^1,2^ The World Health Organization (WHO) has reported that village health workers (VHWs) can enable health systems to achieve significant progress in improving the effectiveness and efficiency of PHC services through increased access to preventive and promotive services such as early diagnosis and treatment of minor conditions.^3^

Village health workers help to bring services closer to the communities and are effective in fostering positive behaviour change through social connectedness, building trust and reducing stigma.^4^ Their services have been shown to improve the relevance, acceptability and accessibility of PHC services through home visits, assessment and treatment of minor health ailments and referrals for complicated infirmities.^5^ The engagement of VHWs has also been shown to improve the productivity of PHC through task shifting from qualified physicians and nurses to less specialised communities.^6^

Community health aims to prevent diseases and promote healthy living through an emphasis on reaching people who experience the greatest burden of both acute and chronic disease, disability and death.^7,8^ Sustainable, effective and efficient delivery of community health services has been a challenge in poorly resourced countries which rely on VHWs for PHC provision.

As countries in the Global South continue to be affected by the shortage of health care workers because of migration to richer Western countries, there has been a renewed interest in the engagement of VHWs to bridge this gap.^9^ Zimbabwe reportedly had 8 core health workers per 10 000 population in 2015 against the WHO’s recommended 23 per 10 000 population.^9^ HIV/AIDS and migration have been cited as the main contributory factors for health care worker shortages.^9,10^ Low-income countries, including Zimbabwe, are forced to rely on the services of VHWs to deliver essential PHC services. The VHW programme in Zimbabwe was found to be ineffective in supporting the rural health centres and clinics for promotive, preventive, curative and rehabilitative services at household level under the context of PHC.^11^ Poor health outcomes have continued to characterise the health system with the infant mortality rate (IMR) increasing from 53 per 1 000 in 1992 to 56 per 1 000 live births in 2016 and the maternal mortality rate (MMR) at 443 per 100 000 which is a way too high in relation to the SDG target of 70 per 100 000.^12^

The situational analysis in Zimbabwe revealed a lack of a standardised package of health services which are offered by the VHWs which holistically address the current and emerging health needs of the communities. Attempts to improve the effectiveness and efficiency of such programmes have seen these not being fully operationalised in various settings because of a lack of clear-cut implementation strategies.^11^

If community health system strategies are not put in place to improve the effectiveness and efficiency of the VHWs in service delivery, the national health systems could continue to be overstrained.^3^ This could impact negatively on the sustainability of community-based health care in the villages such as regular HIV testing, counselling, TB case finding and management, and increased chronic illness treatment default rate in the remote villages.^10^ Furthermore, ineffective community health systems could significantly contribute to ever increasing incidence and prevalence of communicable diseases such as malaria, HIV, TB and an increased frequency of cholera outbreaks in such countries.^12^

A strategy has generally been defined as the determination of basic long-term goals and objectives of an enterprise and courses of action including resource allocation to achieve that goal.^13^ In this study, a strategy is a plan of action designed for the VHWs to deliver effective and efficient PHC services. A VHW, used synonymously with community health worker (CHW), is a lay health care worker who has received standardised training outside the normal medical curricula and has a defined role in the delivery of PHC.

There is a need to clarify the concept of effective and efficient strategy for VHWs in the delivery of PHC services. The WHO emphasises the importance of clarification and having frameworks to guide the delivery of effective and efficient PHC by health care staff.^14^ This study sought to map the literature on the health system strategies that can be used to improve the effectiveness and efficiency of VHWs in PHC delivery as guided by Rodgers’ Evolutionary Concept Analysis. The study also aims to leverage the findings to develop a conceptual framework that guides the research on ‘Strategies to improve the effectiveness and efficiency of VHWs in service delivery in a selected district of Zimbabwe’.

## Methods and design

### Study design

A scoping review was used to map and provide an overview of the existing literature from 2010 to 2022 on the strategies for effective and efficient delivery of PHC by VHWs. Scoping reviews are essential to determine the gaps in the existing and emerging literature for future research such as systematic reviews that would need to be undertaken on the broad topics.^15^

### Conceptual framework

Rodgers’ Evolutionary Conceptual Framework was used to analyse the concept of ‘Empowering VHWs with effective strategies and support is key to unlocking healthier future for all’. According to this framework, concepts are dynamic, relative and contextual.^16^ This method also emphasises that description and clarification of the concept are foundational for ongoing concept development and further research. Three elements of the conceptual framework are distinguishable: antecedents, attributes and consequences.^17^ Antecedents are the events that must occur before the concept can occur and are influenced by internal, external and environmental factors of the health system.^17^ Various factors which involve health system, community and VHW-related were found to influence the concept of effective and efficient strategies in PHC service delivery by VHWs. Attributes are the characteristics which help clarify the conceptual meaning in context. The consequences are the outcomes of effective and efficient PHC services as delivered by the VHWs.^18^

### Data collection and analysis

#### Inclusion criteria

The review included studies that reported PHC service delivery strategies by VHWs/CHWs. Only studies, reports and articles presented in English from January 2010 to December 2022 in peer-reviewed journals. Qualitative, quantitative or mixed methods research and reports obtained from PUBMED, EBSCO, ScienceDirect and Google Scholar databases were reviewed. The authors did not consider the use of grey literature as they felt that these had not undergone the rigorous peer review process.

#### Exclusion criteria

The review excluded studies on other health systems outside PHC delivery services.

### Data sources and search strategy

The scoping review utilised the PUBMED, EBSCO, ScienceDirect and Google Scholar databases to generate articles for review in accordance with the inclusion criteria. Search terms included: ‘village health workers strategies’ OR ‘community health worker strategies’ OR ‘Lay-health worker strategies’ OR ‘effective delivery of primary health care by village health workers’ OR effective delivery of PHC by community health workers’ OR ‘effective delivery of PHC by Lay- health workers’ OR ‘efficient delivery of PHC by village health workers’ OR ‘efficient delivery of PHC by community health workers’ OR ‘efficient delivery of primary health care by Lay- health workers’.

#### Review methods

Titles and abstracts were reviewed by the researcher and checked by the two co-promoters to identify and screen articles that would be relevant to the systematic review. Full texts of articles that met the inclusion criteria were further reviewed. Disagreements and differences were resolved by dialogue until a common ground was found. A Mendeley software’s duplicate checking tool was used whereby the reference sets were first expanded and merged. A manual check was then thoroughly used to check for any possible duplicates in the folder with all the references followed by an alphabetical ranking of the title columns.

#### Data extraction

The articles that met the inclusion criteria had Rodgers’ Evolutionary Conceptual Analysis Framework applied to antecedents, attributes and consequences for PHC delivery by VHWs and/or CHWs. An MS Excel spreadsheet was collaboratively developed in line with this framework to ensure consistent and homogenous data collection criteria by the researcher and the two co-promoters. Data collected were compared and any inconsistencies dialogue to reach a consensus between the researcher and the two co-promoters.

#### Data analysis

The findings from the reviewed articles that met the inclusion criteria were qualitatively analysed in line with Rodgers’ Evolutionary Concept Analysis to identify and explain antecedents, attributes and consequences on the delivery of PHC systems by the VHWs. During analysis, tentative codes were created and iteratively modified by the research team. The developed codes were reviewed to show how they relate to each other and to identify the patterns. Categories of data sets were created which finally led to the development of the themes which were finally analysed in terms of the antecedents, attributes and consequences (Appendix 1).

#### Quality assessments

We used the Preferred Reporting Items for Systematic Reviews extension for Scoping Reviews (PRISMA-ScR) to ensure rigour and provided guidance on knowledge synthesis in line with Rodgers’ Evolutionary Conceptual Analysis Framework. The checklist contained 20 essential reporting items and two optional items (Appendix 2). Synthesised evidence was mapped in a graph and tables.^19^

### Ethical considerations

Ethical clearance to conduct this study was obtained from the University of Venda Research Ethics Committee (reference no.: FHS/23/PH/11/0709).

## Results

**FIGURE 1:**
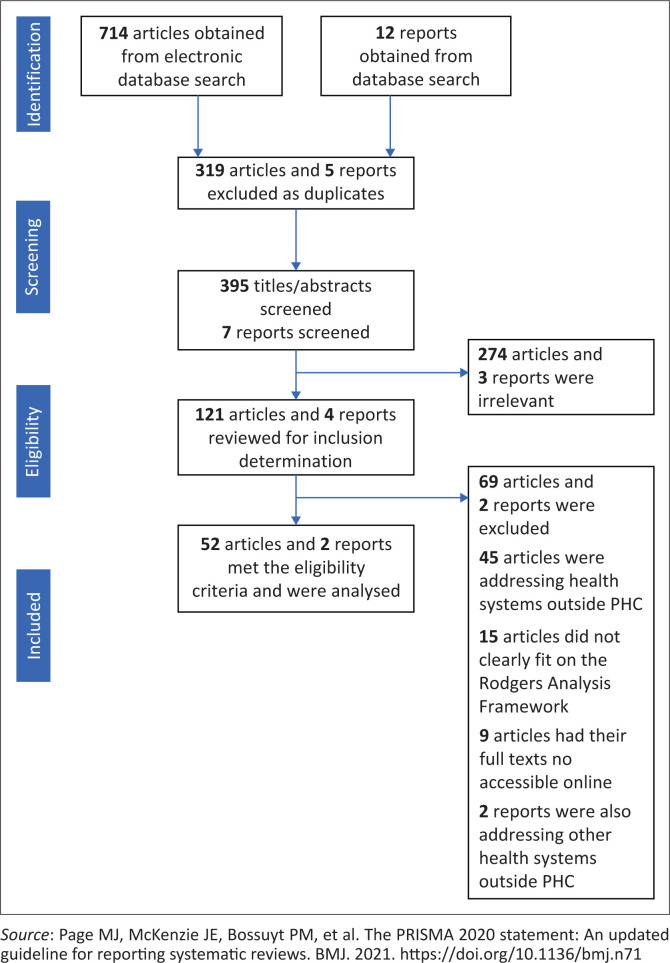
PRISMA flowchart.

There were 714 articles and 12 reports obtained from the searched databases. After removing duplicates, 395 articles and seven reports remained. These had their abstracts reviewed in line with inclusion criteria by the research team leading to the elimination of 274 articles and three reports. The articles and reports that met the inclusion criteria had their full text studied by the three researchers leading to 52 articles and two reports as summarised on the PRISMA flow chart (Figure 1).

### Antecedent, attributes and consequences of village health worker strategies

#### Antecedents

Effective and efficient PHC delivery strategies by VHWs were synthesised and categorised into three broad themes: health system, organisational, and VHWs-related and community-related (Table 1).

**TABLE 1:** Antecedents.

Antecedent	Supporting literature
Enabling work environment	Refs. 6, 20, 21, 22
Community engagement	Refs. 4, 23, 24
Motivation	Refs. 25, 26, 27
Incentives	Refs. 5, 6
Community health integration into the mainstream health system	Refs. 28, 29, 30, 32, 31, 33
Use of information and communication technology (ICT)	Refs. 20, 21, 34, 35, 36

Note: Please see full reference list of this article: https://doi.org/10.4102/phcfm.v16i1.4554.

**Enabling work environment:** Working conditions are a function of the broader human resource management for the creation of a decent work environment, boosting morale and reducing staff attrition.^20^ The enabling work environment for the VHWs should provide the inputs required and suitable working conditions. Such elements include sustainable workload, adequate supplies and equipment.^20,21^

Optimal tasks are therefore important as too many tasks can overwhelm the CHWs and impact adversely on the quality-of-service delivery and ineffectiveness of the programme.^21^ Unsustainable workload was found to have contributed to the neglect of essential tasks by the Lay Health Workers in Malawi and Pakistan.^6^ According to the Global Health Workforce Alliance,^22^ the elements of an enabling work environment for VHWs are influenced by the following: context (political, socio-cultural and economic environment); actor-based (state, civil society and private sector); relational in terms of power dynamics between VHWs and the professional health workers as actors in the health system; and health system oriented as determined by ‘whole of system’ building blocks perspective.^22^

**Community engagement and participation:** Community engagement is an essential component of any health system strategy to achieve its goals. It is a process of developing relationships for stakeholders to work together with the VHWs for a common health cause, it can enable changes in behaviour, practices, environment, policies and programmes of the local communities.^23^ Five levels of community engagement have been outlined as follows: information provision; consultation; community decision-making through consensus; collaboration and empowerment.^23^ Local communities ought to be involved in the selection of the VHWs and be part of the programme design, implementation and monitoring. In Nigeria, the formation of village health committees (VHCs) was one of the various strategies used to effectively improve the delivery of PHC services by the VHWs.^24^

There was collaboration with the local communities which started with their selection of CHWs and also taking part in programme planning, monitoring and evaluation using locally obtained health-related data through participatory rural health appraisals.^24^ Local communities were found to have ownership of the programme and its sustainability was enhanced through the formation and support of various women groups with micro-credit schemes for the VHCs.

Under the Nigerian model, some of the older community members were used as role models in health campaigns to promote good health behaviours.^24^

In Malawi, the improved adherence to the treatment regimen for TB and HIV was attributable to the role of the ‘guardians’ who supported the Health Surveillance Assistants (HAS) to observe the patients taking their daily medications. The ‘guardians’ were senior and/or elderly members of the local communities who volunteered to support the activities of the CHWs in Malawi.^4^ In Guinea, the local communities were involved in the selection and monitoring of the CHW’s activities such that they value them as community health liaisons. The latter informs their local communities about the impending outreach clinic dates and in turn encourages men and women to attend health education sessions. The communities greatly support these cadres in finding the loss of follow-up patients leading to improved treatment outcomes for those with chronic ailments.^23^

**Motivation:** Motivation has been regarded as a phenomenon that is a product of psychological, interpersonal and contextual factors.^25^ It was further qualified as that which relates to the intrinsic and extrinsic factors that account for VHWs to exhibit direction, persistence and intensity of efforts towards achieving health goals.^26,27^ Intrinsic factors comprise the psychological needs for organisational recognition and growth encompassing career advancement and increased responsibility for higher-level tasks for the VHWs. Extrinsic motivational factors for the VHWs are externally imposed on the individuals and include rewards like money, grades, competition and coercion among others.^26^ Direct incentives for CHWs can be both financial and non-financial while on the contrary indirect ones are mostly health systems provided.^25^

**Incentives:** Performance-based incentives were found to enhance the effectiveness of CHWs service delivery in different settings. In India, CHWs, also known as Accredited Social Health Activists (ASHAs) were highly motivated in delivering PHCs through the provision of both performance-based incentives and life assurance as a reward for job performance. In Rwanda, their performance-based system was based on indicators for nutrition, antenatal care, facility delivery, TB and HIV control among others. In the latter, a third of the payment goes to the individual CHW while the remainder to the cooperative to encourage both individual and team performance.^25^ In South Africa, the CHWs were provided with scholarships for career progression as a motivational factor while in Madagascar and Malawi, CHWs cadres can advance to supervisory positions in the community health service.^6,25^

**Community health integration into national health systems:** For VHWs to be effective in the delivery of PHC service, there should be support from the mainstream health system in terms of the provision of logistical and drug supplies.^28^ For this to be achieved, there should be a deliberate government policy to ensure all the CHW programmes are aligned with it.^28^ Several emerging economic countries such as Brazil, South Africa, India and Pakistan have integrated and institutionalised CHWs into the mainstream health system^29^ to address fragmentation and uncoordinated systems, promote community utilisation of PHC services, and address large inequities in the access and coverage of health care services.^30^

Brazil’s Unified Health System (SUS) launched its National Community Health Worker Programme in 1991 followed by the Family Health Strategy in 1994 and an Act of Parliament in 2002 to regulate CHW recruitment being part of the evolutionary process.^31^ The SUS provides for CHWs working as part of either Community Health or Family Health Teams where there are members of a group of PHC experts encompassing nurses, nurse aides, physicians and family health doctors.^32^ This integration and policy support contributed to the doubling of immunisation coverage to 98%, and reduced infant and maternal mortality rates by 75% and 58% (respectively) in 2000.^30^ These were achieved through a holistic model which addresses social determinants of health such as sanitation and hygiene, education and healthy public policies. These models were found to be cost-effective with higher levels of customer satisfaction,^30,33^

**Use of information and communication technology:** The globalised world has seen an evolution from paper-based to digital data collection and monitoring of trends through the use of cell phones and tablets. The practice of medicine and public health supported by mobile devices is popularly known as mHealth. This technology rapidly connects CHWs with their supervisors and clients. It can reduce delays in patient care and enhance managerial and supervisory decisions required for the day-to-day functioning of the PHC system.^20,21^ This system continues to develop in line with the advancement in technology and has now spread to developing countries. In Uganda, CHWs used cell phones to send real-time data on rural HIV-positive patients by tracking and monitoring clinical and drug adherence.^34,35^ In Ghana and Zambia, mHealth was effectively used by the CHWs for primary data collection and monitoring of rapid diagnostic test (RDT) kits for malaria and also for validation of information. Rwanda used the UNICEF-developed Rapid SMS system for enhanced communication and alerts which led to the improved outcomes of MCH.^36^

#### Attributes

Table 2 describes the attributes of village health worker strategies.

**TABLE 2:** Attributes.

Attribute	Supporting literature
Ongoing training and skills development	Refs. 20, 37, 38, 39, 40, 41
Mutual respect and trust between VHW and communities	Refs. 42, 43
Supportive supervision	Refs. 20, 21, 44, 45, 46
Enhanced contact between VHWs and communities	Refs. 46, 47
Teamwork	Refs. 48, 49, 50

Note: Please see full reference list of this article: https://doi.org/10.4102/phcfm.v16i1.4554.

VHW, village health worker.

**Ongoing training and skills development:** The WHO provided that CHWs should timeously receive ongoing training and refresher courses. While different settings have variations on the frequency, content and structure of ongoing training and refresher courses, the United States Agency for International Development (USAID) Health Care Improvement Project has recommended that these should be provided at least once in 6 months to aid in the development of new skills.^37^ The importance of ongoing training and skill development has been necessitated by the ever-changing roles of CHWs which shifted from traditional health promotion, education and linking the communities to the health system to curative.^38^ Through task shifting, the CHW’s roles now include management of mental health, disease surveillance, diagnosis and treatment of existing and emerging pandemics and following the integrated community case management (iCCM) standard guidelines.^38^

Various studies in different places have reported health-related positive changes in behaviours, attitudes and practices among CHWs who received more frequent refresher training.^20, 38, 39^ For instance, children managed by CHWs who received refresher training in terms of iCCM were more likely to have better outcomes than those who were not.^39^

Participatory action research was recommended by the WHO when designing refresher training programmes for CHWs, as these could provide them with the autonomy to seek the support they require to effectively deliver quality PHC and stay motivated.^40, 41^ Training should be tailored to suit the local context in terms of content, baseline competencies and expected roles and should balance theory and practical skills.^41^

**Mutual respect and trust between village health workers and communities:** The participation of local communities in the community health system is characterised by mutual respect and joint ownership and sustenance of such programmes. Community involvement in the selection and performance monitoring and evaluation of the CHWs was found to be an important enabler of community health programme effectiveness as it is associated with the retention, motivation, performance, accountability, acceptability and support of the uptake of the CHWs’ work.^42^ Local communities’ trust in the services of the CHWs relates to how effective, efficient and responsive they are to their health preferences. The more the CHWs spend time living and engaging with their local communities on PHC services, the more the satisfaction of the clients on the services which they provide. The level of trust shown by the local communities in the services delivered by CHWs is anchored by the professional and ethical conduct of the CHWs.^43^

**Supportive supervision:** Supervision for CHWs has evolved from task oversight and punitive with critical corrective action to supportive/facilitatory.^21,44,45^ Supportive supervision comprehensively necessitates collaborative reviews, observation, monitoring, constructive feedback and problem-solving.^44,45^ Effective and efficient delivery of PHC services can be enhanced by supportive supervision which creates an enabling environment which links CHWs and the health system.^46^ Kok et al. also found that supportive supervision can contribute to improved health outcomes.^46^ There is a need to sustain the supportive supervision programme through the provision of financial resources and time to ensure a gradual shift in behaviour and attitude through supervisor training on communication approaches, team building and promoting lasting relationships with their communities.^20,44^

Supervisors should ensure the credibility of the VHWs within their communities through role clarification, ensuring adequate supplies and solving health-related problems.^45^ Effective supportive supervision systems should have its objectives clarified in terms of: (1) quality assurance (adherence to norms and guidelines, drug and equipment supplies); (2) supportive environment (emotional, coaching and community problem solving) and (3) communication and information (collection of data on household visits, expanded programme on immunisation (EPI) coverage and deliverance of promotional messages).^21^

The most effective supportive supervisory assessments were: (1) group supervision which focusses on goal setting and problem-solving; (2) engagement of stronger peers to support weaker ones through job training and mentorship; (3) community monitoring of CHW performance and (4) onsite visit from the supervisors and scheduled CHWs self-assessments and regular mentor to mentee phone calls.^21^

A mixed method study by Kok et al.^45^ evaluated a group supervision intervention in Mozambique, Malawi, Ethiopia and Kenya which included training and mentorship of supervisors and found improved motivation and increased empowerment and participation of CHWs in decision-making. The participants also found value in the process of supervision, problem-solving focussed on joint responsibility and teamwork, learning and skills sharing and the facilitatory role of the supervisor. These authors recommended a combination of groups with individual, or peer supervision coupled with CHW performance assessment and feedback mechanisms.^46^

**Enhanced contact between village health workers and communities:** Personal contact between the CHWs and communities can improve both the effectiveness and efficiency of PHC delivery. Enhanced personal interactions between communities and CHWs can lead to positive engagements through establishing a long-lasting relationship and participation in health issues.^46^ In a bid to reduce maternal and neonatal mortality rates through increased personal contact between CHWs and communities, Nigeria’s health system used VHWs who were expected to visit at least 30 households per month on MNCH delivery.^47^ The VHWs had the most direct personal interactions with the community members in their homes as compared to the other models. The outcomes from the VHWs were such that the women who were directly interacted with were two times more likely to know the MNCH danger signs and adopt the recommended preventive and control measures. This was comparable to the other two models which had Community Volunteers (CVs) and Junior Community Health Extension Workers (JCHEWs) who conducted community group discussions on the same topics in a central location.^47^

**Teamworks:** Teamwork enhances the delivery of effective and efficient PHC services by the VHWs.^48^ Strategies for team building include: VHW cadres keeping each other’s contacts; regular meetings and planning for outreach sessions together; development of common understanding of the state of community health care programmes; assisting each other in adapting health messages to their local contexts; and constructive and supportive supervision.^49^ Further emphasis was put on shared goals, role clarity and effective communication which enables improved coordination of care and efficient use of health care resources contributing to enhanced community satisfaction with care and acceptance of treatment.^48^ A study in South Africa revealed that teamwork nurtured positive attitudes among the CHWs leading to the effective delivery of PHC services.^50^

#### Consequences

Table 3 outlines the consequences of village health worker strategies.

**TABLE 3:** Consequences.

Consequence	Supporting literature
Equitable community access to essential PHC services	Refs. 51, 52
Improved quality of PHC delivery	Refs. 51, 53, 54

Note: Please see full reference list of this article: https://doi.org/10.4102/phcfm.v16i1.4554.

PHC, primary health care.

**Equitable access to essential primary health care services:** Many scholars have established that CHWs promote equitability in access and utilisation of PHC services regardless of the place of residence, gender, educational level or socio-economic status.^51,52^ Well-strategised CHW programmes can eliminate barriers faced by those in need of health care at both the community and at PHC facilities. In Ghana, the Navrongo Community Health and Family Planning Project (popularly known as the Navrongo Experiment) was globally regarded as one of the successful PHC deliveries by the CHWs. The project was reportedly aimed at bridging the gap between urban and rural dwellers by improving access to PHC in deprived communities using community-driven resources and structures. The strategies were devised for CHWs to provide community outreach and mobilisation for health promotion and referral linkages to the PHC facilities. After 5 years, an outcomes evaluation of the strategies employed revealed increased access to health care services and immunisation coverage with a 50% reduction in maternal and child mortality rates and a 15% fall in the fertility rate.^51^ The improved overall rural population health was also attributable to improved equity in PHC delivery for the rural communities.^51^

**Improved quality of primary health care delivery:** Increased access and utilisation of PHC services by the local communities coupled by an efficient referral system can lead to improved quality of health outcomes.^53^ The health systems of Sri Lanka, India and Indonesia supported CHWs to deliver effective health promotional, basic curative services and child immunisation services coupled with effective referral linkages in the remote rural areas leading to decreased maternal and neonatal mortalities.^53^ Despite widespread health worker attrition and lack of a framework to guide CHWs’ service delivery operations, Ghana enrolled and utilised CHWs to help eradicate Guinea worm and increased coverage of the EPI and improved adherence to HIV management.^51^ The CHWs in sub-Saharan Africa are involved in ICCM where they have been trained to assess, classify and treat pneumonia, malaria and diarrhoea. Integrated Community Case Management has reportedly contributed to the declining cases of complicated pneumonia and malaria by as much as 76%.^54^

#### Development of a conceptual framework

**FIGURE 2:**
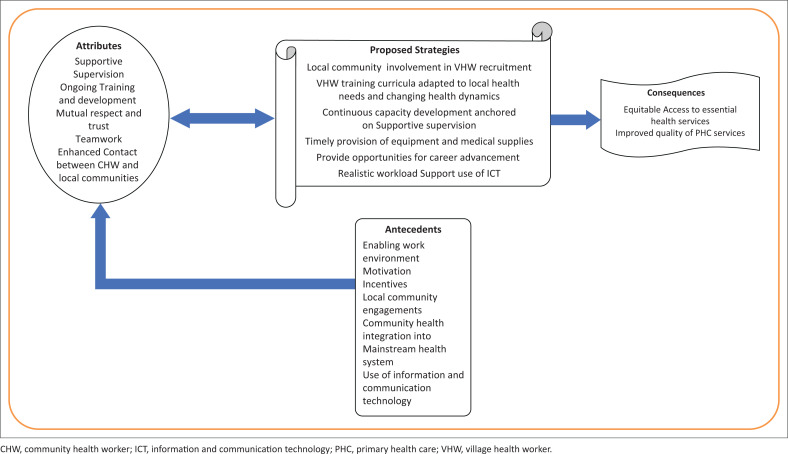
Framework using findings from Rodgers’ Evolutionary Conceptual Analysis.

The outcomes of the reviewed literature informed the development of a conceptual framework from the clarified concept of ‘Empowering VHWs with effective strategies and support is key to unlocking healthier future for all’. Antecedents were identified and the related attributes which influenced the consequences of effective and efficient PHC service delivery by the CHW/VHWs. The theoretical framework has been presented in Figure 2.

### Strategies to enhance service delivery for village health workers

- Recruitment of VHWs in accordance with appropriate selection criteria such as literacy, self-motivation, gender and local resilience and as determined by contextual factors
- Adapt VHW training curricula to the local health needs using appropriate training methodologies applicable to adult learning and ensure continuous evaluation to improve the learning materials
- Ensure a sustained continuous capacity development for VHWs in the form of programmed and ad hoc refresher training augmented with regular supportive supervision
- Align the VHW with the perspective of the community and the role of other health care cadres to optimise integrated PHC services to promote intersectoral coordination and synergy with the wider health system
- Supply equipment and job aids necessary to deliver the necessary PHC services
- Motivation of VHWs with both financial and nonfinancial incentives
- Establish effective linkages and a referral system
- Establish appropriate indicators to monitor and track the performance of individual VHWs and the community health programme in general
- Determine appropriate and realistic scope of work that addresses priority community health service gaps.

## Discussion

The nomenclature of CHWs is differentiated by context and setting but is commonly known as VHWs, Lay-health workers and CVs. The universal definition of CHW/VHWs refers to lay health workers who received standardised training outside the usual medical curricula.^37^ Community health is delivered under the confines of PHC and VHWs are now an integral part of this system.^28^ The antecedents which enable the effective and efficient delivery of PHC services by VHWs include: the provision of an enabling environment, community health integration into the mainstream health system, community engagements, motivation, incentives and information communication technology.

The provision of an enabling and decent work environment for VHWs can involve developing new or reorienting existing policies to ensure their effective and efficient delivery of PHC services. It has been established that VHWs generally feel they are under-resourced to efficiently deliver health services which are responsive to the needs of their communities.^28^

Olaniran et al.^55^ concurred with the previous scholars having revealed that PHC service coverage by CHWs is hampered by inadequate supplies and equipment, lack of transport and limited infrastructural support. Various studies emphasise the optimal tasks for individual VHWs. In literature, there has not been a universally agreed optimum number of tasks for the individual VHW. The Community Health Worker Assessment and Improvement Matrix (CHW AIM) has provided that VHW tasks should be centred on health promotion for disease prevention and control, testing and curing for endemic infirmities, referrals, family planning and visits to client homes.^37,47^

There has been a drive to integrate community health into mainstream health systems in low- and middle-income countries. Some scholars are in agreement that unreliable funding hinders the mission of integration. Moreso, they maintained that there is limited professional recognition of CHWs’ potential owing to a lack of consistency in their selection criteria, training, scope of practice, workload and outcome measures.^55,56^

It has been widely agreed that for CHWs to provide effective and efficient services, some form of motivation is required. Persistent challenges have been faced by health systems to encourage community members to enrol as VHWs who are volunteers and then to motivate them to perform their work effectively and sustainably over time.^25^ Most of the community health programmes in low-income countries are not so effective as these cadres are often regarded as volunteers rather than full-time employees of the health system and consequently, they do not really enjoy the protection of the labour laws in their contexts. Some scholars are in agreement that opportunities for career advancement and social recognition and respect from the health system and communities seem to motivate the VHWs to effectively deliver PHC services as might be compared with financial incentives.^27,28^

Ongoing and refresher training are vital for the VHWs to be effective. However, many of such programmes fail as their design is not contextually aligned.^42,57,58^ Socio-cultural sensitivity is vital in settings which are dominated by traditional medicine practices and should consider incorporating the natives to be part of the facilitators for the training programmes.^10,59^

The use of ICT in PHC service delivery by the VHWs continues to be encouraged in line with globalisation and technological advancement. Many scholars agree that the technology seems to be effective in many settings with real-time data collection and transmission. However, on social considerations, Campbell et al.^34^ and Twimuky et al.^35^ found that some patients were particularly not entirely content with the technology as this subject to some kind of stigma and that in some settings, the programme had some complex technical issues such as network outages and system failure. It has also been argued that ICT in health service delivery brings with it as many opportunities as challenges.^28^ It was explained that while low- and middle-income countries have embraced the technology to efficiently deliver PHC services, most of these programmes fail at the implementation stage. The failures were attributable to human and contextual factors such as lack of ICT competence, socio-cultural contexts, and lack of funding and poor technical support to the ICT programme.^28,60^

The evolution of supervision to supportive supervision has been associated with improved delivery of PHC services by the CHWs through team building, mentorship, motivation and identification of training needs and adherence to standards which contributes to improved treatment outcomes.^44,45,50^ This system was also found to have challenges in terms of costs of implementation and sustainability leading to it being done on an irregular basis and generally lacking in feedback.^61^ In many developing countries such as Kenya and Benin, there is generally a lack of logistical support in terms of vehicles, fuel and per-diems for supervisors.^20^ Some scholars also argue that the health outcome improvements have not been attributable to supportive supervision outside other interventions or contextual changes in the health system.^46^

### Limitations of the study

The articles were collectively reviewed along thematic lines as informed by the Rodgers’ Analytical Framework rather independently. This could have led to a bias towards themes rather than in-depth findings of each individual article.

### Implications

This scoping review mapped the existing literature on the strategies that can be used to improve the effectiveness and efficiency of VHWs in the delivery of PHC using Rodgers’ Evolutionary Conceptual Analysis. We recommend a comprehensive systematic review of the literature and other empirical studies to be carried out to determine and evaluate such strategies in different settings and contexts.

## Conclusion

The PHC services delivered by VHWs have evolved from being an extension of the health system to becoming an integral part of the mainstream health system. This evolution is evidenced by the task shifting and task sharing that nurses and VHWs undertake for minor diagnostic and curative services in low- and middle-income countries. The focus is now on reorganising community health programmes to ensure efficient and responsive PHC service delivery by VHWs through their integration into the mainstream health system. The engagement of local communities in VHW selection, programme design and implementation, as well as monitoring and evaluation, is crucial for the effective and efficient delivery of PHC services and for ensuring the sustainability of community health activities. The growing demand for effective services from VHWs has led to a shift from volunteerism towards full-time employment, considering motivational aspects.

Effective and efficient PHC services offered by VHWs necessitate teamwork. Such systems are characterised by supportive supervision programmes that ensure constructive criticism and collective problem-solving. Strategies for delivering PHC services effectively and efficiently should be informed by the context and aligned with a dynamic environment, taking ICT into account.

## Appendices

**Appendix 1:** Summary of included articles and data extraction sheet.

Author	Year	Title	Study design	Antecedents	Attributes	Consequences
Ndambo et al.^4^	2022	The role of community health workers in influencing social connectedness using the household model: A qualitative case study from Malawi	Case Study	Community engagement	-	-
Braun et al.^5^	2013	Community Health Workers and Mobile Technology	Commentary	Incentives Use of Information and Communication Technology	-	-
Smith et al.^6^	2014	Task-shifting and prioritization: A situational analysis examining the role and experiences of community health workers in Malawi	Qualitative (focused group discussions and interviews)	Enabling work environment Incentives	-	-
Perry et al.^20^	2014	Developing and Strengthening Community Health Worker Programs at Scale A Reference Guide and Case Studies for Program Managers and Policymakers	Case Study	Enabling work environment Use of Information and Communication Technology	Ongoing training and skills development Supportive supervision	-
Crigler et al.^21^	2011	Developing and Strengthening Community Health Worker Programs at Scale: A Reference Guide and Case Studies for Program Managers and Policy Makers	Case Study	Enabling work environment Use of Information and Communication Technology	Supportive supervision	-
Global Health Workforce Alliance^22^	2010	Integrating Community Health Workers in national health workforce plans	Commentary	Enabling work environment	-	-
Kok et al.^23^	2017	Optimising the benefits of community health workers’ unique position between communities and the health sector: A comparative analysis of factors shaping relationships in four countries	Qualitative comparative study	Community engagement	-	-
Abdulraheem et al.^24^	2012	Primary health care services in Nigeria: Critical issues and strategies for enhancing the use by the rural communities	Qualitative	Community Engagement	-	-
Colvin^25^	2014	What Motivates Community Health Workers? Designing Programs that Incentivize Community Health Worker Performance and Retention. Developing and Strengthening Community Health Worker Programs at Scale: A Reference Guide and Case Studies for Program Managers and Policy Makers	Case Study	Motivation	-	-
Aduo-Adjei et al.^26^	2016	The Impact of Motivation on the Work Performance of Health Workers (Korle Bu Teaching Hospital): Evidence from Ghana	Qualitative	Motivation	-	-
Sarriot et al.^27^	2021	Motivation and Performance of Community Health Workers: Nothing New Under the Sun, and Yet	Commentary	Motivation	-	-
Austin-Evelyn et al.^28^	2017	Community health worker perspectives on a new primary health care initiative in the Eastern Cape of South Africa	Focus group discussions and surveys	Community health integration into mainstream health system	-	-
Krieger et al.^29^	2022	How do community health workers institutionalise: An analysis of Brazil’s CHW programme.	Case Study	Community health integration into mainstream health system	-	-
Machado et al.^30^	2019	Family and community medicine in the supplementary health system in Brazil: Implications for the Unified National Health System and for physicians	Case Study	Community health integration into mainstream health system	-	-
Minayo et al.^31^	2018	A brief history of worker’s health in Brazil’s unified health system: Progress and challenges	Qualitative descriptive study	Community health integration into mainstream health system	-	-
Pinto et al.^32^	2012	Community Health Workers in Brazil’s Unified Health System: A framework of their praxis and contributions to patient health behaviours	Qualitative descriptive survey	Community health integration into mainstream health system	-	-
Johnson et al.^33^	2013	Learning from the Brazilian community health worker model in North Wales	Commentary	Community health integration into mainstream health system	-	-
Campbell et al.^34^	2018	Ugandan Study Participants Experience Electronic Monitoring of Antiretroviral Therapy Adherence as Welcomed Pressure to Adhere	Qualitative exploratory	Use of Information and Communication Technology	-	-
Twimukye et al.^35^	2021	Acceptability of a mobile phone support tool (Call for Life Uganda) for promoting adherence to antiretroviral therapy among young adults in a randomized controlled trial: Exploratory qualitative study	Qualitative exploratory	Use of Information and Communication Technology	-	-
Ngabo et al.^36^	2012	Designing and Implementing an Innovative SMS-based alert system (RapidSMS-MCH) to monitor pregnancy and reduce maternal and child deaths in Rwanda	Qualitative exploratory	Use of Information and Communication Technology	-	-
Crigler et al.^37^	2011	Community Health Worker Assessment and Improvement Matrix (CHW AIM): A Toolkit for Improving CHW Programs and Services.	Case studies	-	Ongoing training and skills development	-
Schleiff et al.^38^	2021	Schleiff MJ, Aitken I, Alam MA, Damtew ZA, Perry HB. Community health workers at the dawn of a new era: Recruitment, training, and continuing education	Qualitative exploratory	-	Ongoing training and skills development	-
Wanduru et al.^39^	2016	The performance of community health workers in the management of multiple childhood infectious diseases in Lira, northern Uganda – A mixed methods cross-sectional study	Mixed methods	-	Ongoing training and skills development	-
Tetui et al.^40^	2017	A participatory action research approach to strengthening health managers’ capacity at district level in Eastern Uganda	Qualitative	-	Ongoing training and skills development	-
Carboni et al.^41^	2022	Training-of-trainers program for community health workers involved in an innovative and community-based intervention against malaria among goldminers in the Guiana shield: A quality and effectiveness evaluation	Mixed methods	-	Ongoing training and skills development	-
Vanden et al.^42^	2022	Understanding Trustful Relationships between Community Health Workers and Vulnerable Citizens during the COVID-19 Pandemic	Evaluation Qualitative	-	Mutual respect and trust between VHW and communities	-
Sripad et al.^43^	2021	Measuring client trust in community health workers: A multi-country validation study	Mixed methods	-	Mutual respect and trust between VHW and communities	-
Kangovi et al.^44^	2015	From Rhetoric to Reality – Community Health Workers in Post-Reform U.S. Health Care	Commentary	-	Supportive supervision	-
Kok et al.^45^	2018	Does supportive supervision enhance community health worker motivation? A mixed-methods study in four African countries	Mixed methods	-	Supportive supervision Enhanced contact between VHWs and communities	-
Findley et al.^47^	2017	Cost-Effectiveness of Alternative Models of Community Health Workers for Promotion of Maternal, Newborn and Child Health in Northern Nigeria	Quasi-experimental	-	Enhanced contact between VHWs and communities	-
Babiker et al.^48^	2014	Health care professional development: Working as a team to improve patient care	Qualitative descriptive	-	Teamwork	-
Mishra^49^	2014	Trust and teamwork matter’: Community health workers’ experiences in integrated service delivery in India	Qualitative Ethnographic	-	Teamwork	-
Tseng et al.^50^	2019	Integrating community health workers into the formal health system to improve performance: A qualitative study on the role of on-site supervision in the South African programme	Case Study	-	Teamwork	-
Baatiema et al.^51^	2016	Community health workers in Ghana: the need for greater policy attention	Qualitative Ethnographic	-	-	Equitable community access to essential PHC services Improved quality of PHC delivery
Tuyisenge et al.^52^	2019	Facilitating equitable community-level access to maternal health services: Exploring the experiences of Rwanda’s community health workers	Case Study	-	-	Equitable community access to essential PHC services
Sarfraz & Hamid^53^	2015	Challenges in delivery of skilled maternal care – experiences of community midwives in Pakistan	Qualitative Focused Group Discussions	-	-	Improved quality of PHC delivery
Amouzou et al.^54^	2014	Where is the gap?: The contribution of disparities within developing countries to global inequalities in under-five mortality	Descriptive Quantitative survey	-	-	Improved quality of PHC delivery
Olaniran et al.^55^	2022	Not knowing enough, not having enough, not feeling wanted: Challenges of community health workers providing maternal and newborn services in Africa and Asia	Qualitative Focused group discussions Interviews	Enabling work environment Community health integration into mainstream health system	-	-
Kulbok et al.^56^	2022	Integrating community health workers into mainstream health systems through partnerships	Qualitative descriptive	Community health integration into mainstream health system Enabling work environment	-	-
Ploeg et al.^57^	2019	Contextual factors influencing the implementation of innovations in community-based primary health care: the experience of 12 Canadian research teams	Qualitative Evaluation	-	Ongoing training and skills development	-
Mirzoev et al.^58^	2017	Contextual influences on the role of evidence in health policy development: What can we learn from six policies in India and Nigeria? Evidence and Policy	Qualitative Exploratory	-	Ongoing training and skills development	-
Kwame^59^	2021	Integrating Traditional Medicine and Healing into the Ghanaian Mainstream Health System: Voices From Within	Qualitative Ethnographic	-	Mutual respect and trust between VHW and communities	-
Miiro et al.^60^	2022	Bridging the Gap Between Community Health Workers’ Digital Health Acceptance and Actual Usage in Uganda: Exploring Key External Factors based on Technology Acceptance Model	Mixed methods	Use of Information and Communication Technology	-	-
Ndima et al.^61^	2015	Supervision of community health workers in Mozambique: A qualitative study of factors influencing motivation and programme implementation	Qualitative survey	-	Supportive supervision	-

VHW, village health workers; PHC, primary health care.

**Appendix 2:** PRISMA-ScR checklist.

Section	Item	PRISMA-ScR checklist item	Reported on page #
**Title**			
Title	1	Identify the report as a scoping review	2
**Abstract**			
Structured summary	2	Provide a structured summary that includes (as applicable): Background, objectives, eligibility criteria, sources of evidence, charting methods, results, and conclusions that relate to the review questions and objectives	2
**Introduction**			
Rationale	3	Describe the rationale for the review in the context of what is already known. Explain why the review questions/objectives lend themselves to a scoping review approach	4–5
Objectives	4	Provide an explicit statement of the questions and objectives being addressed with reference to their key elements (e.g. population or participants, concepts and context) or other relevant key elements used to conceptualise the review questions and/or objectives	5–6
**Methods**			
Protocol and registration	5	Indicate whether a review protocol exists; state if and where it can be accessed (e.g. a Web address); and if available, provide registration information, including the registration number	Review protocol not registered
Eligibility criteria	6	Specify characteristics of the sources of evidence used as eligibility criteria (e.g. years considered, language, and publication status) and provide a rationale	7
Information sources*	7	Describe all information sources in the search (e.g. databases with dates of coverage and contact with authors to identify additional sources), as well as the date the most recent search was executed	6–7
Search	8	Present the full electronic search strategy for at least 1 database, including any limits used, such that it could be repeated	7
Selection of sources of evidence	9	State the process for selecting sources of evidence (i.e. screening and eligibility) included in the scoping review	7–8
Data charting process	10	Describe the methods of charting data from the included sources of evidence (e.g. calibrated forms or forms that have been tested by the team before their use, and whether data charting was done independently or in duplicate) and any processes for obtaining and confirming data from investigators	7–8
Data items	11	List and define all variables for which data were sought and any assumptions and simplifications made	6
Critical appraisal of individual sources of evidence	12	If done, provide a rationale for conducting a critical appraisal of included sources of evidence; describe the methods used and how this information was used in any data synthesis (if appropriate)	38–45
Synthesis of results	13	Describe the methods of handling and summarising the data that were charted	8
**Results**			
Selection of sources of evidence	14	Give the numbers of sources of evidence screened, assessed for eligibility, and included in the review, with reasons for exclusions at each stage, ideally using a flow diagram	11
Characteristics of sources of evidence	15	For each source of evidence, present characteristics for which data were charted and provide the citations	12–23
Critical appraisal within sources of evidence	16	If done, present data on critical appraisal of included sources of evidence (see item 12)	38–45
Results of individual sources of evidence	17	For each included source of evidence, present the relevant data that were charted that relate to the review questions and objectives	38–45
Synthesis of results	18	Summarise and/or present the charting results as they relate to the review questions and objectives	12–25
**Discussion**			
Summary of evidence	19	Summarise the main results (including an overview of concepts, themes, and types of evidence available), link to the review questions and objectives, and consider the relevance to key groups	27–29
Limitations	20	Discuss the limitations of the scoping review process.	29
Conclusions	21	Provide a general interpretation of the results with respect to the review questions and objectives, as well as potential implications and/or next steps	30
**Funding**			
Funding	22	Describe sources of funding for the included sources of evidence, as well as sources of funding for the scoping review. Describe the role of the funders of the scoping review	31

*Source*: Adapted from Tricco AC, Lillie E, Zarin W, et al. PRISMA extension for scoping reviews (PRISMAScR): Checklist and explanation. Ann Intern Med. 2018;169(7):467–473. https://doi.org/10.7326/M18-0850

## References

[1] White F. Primary health care and public health: Foundations of universal health systems. Med Prin Pract. 2015;24(2):103–116. 10.1159/000370197 PMC5588212 25591411

[2] Pandey KR. From health for all to universal health coverage: Alma Ata is still relevant. Glob Health. 2018;14(1):1–5. 10.1186/s12992-018-0381-6 PMC6029383 29970118

[3] Scott K, Beckham S, Gross M, et al. What do we know about community-based health programs? A systematic review of existing reviews on community health workers and their integration with health systems. Hum Resour Health. 2018;16(1):16–39. 10.1186/s12960-018-0304-x 30115074 PMC6097220

[4] Ndambo MK, Munyaneza F, Aron M, Makungwa H, Nhlema B, Connolly E. The role of community health workers in influencing social connectedness using the household model: A qualitative case study from Malawi. Glob Health Action. 2022;15(1):2090123. 10.1080/16549716.2022.2090123 35960168 PMC9377265

[5] Braun R, Catalani C, Wimbush J, Israelski D. Community health workers and mobile technology: A systematic review of the literature. PLoS One. 2013;8(6):4–9. 10.1371/journal.pone.0065772 PMC3680423 23776544

[6] Smith S, Deveridge A, Berman J, et al. Task-shifting and prioritization: A situational analysis examining the role and experiences of community health workers in Malawi. Hum Resour Health. 2014;12(1):1–13. 10.1186/1478-4491-12-24 24885454 PMC4014628

[7] Goodman RA, Bunnell R, Posner SF. What is “community health”? Examining the meaning of an evolving field in public health. Prev Med (Baltimore). 2014;67(S1):S58–S61. 10.1016/j.ypmed.2014.07.028 PMC5771402 25069043

[8] Nagel DA, Keeping-Burke L, Shamputa IC. Concept analysis and proposed definition of community health center. J Prim Care Community Health. 2021;12:21501327211046436. 10.1177/21501327211046436 34541950 PMC8460964

[9] Gore O, Mukanangana F, Muza C, Chiweshe M. The role of village health workers and challenges faced in providing primary health care in Mutoko and Mudzi Districts in Zimbabwe in population studies. Glob J Biol Agr Health Sci. 2015;4(1):129–135.

[10] WHO. Community health workers delivering primary health care: Opportunities and challenges. World Health Assembly, 114th session [homepage on the Internet]. 2019 [cited 2022 Jul 22];2018:EB144.R4. Available from: https://apps.who.int/gb/ebwha/pdf_files/EB144/B144_R4-en.pdf

[11] National Community Health Strategy Zimbabwe. National community health strategy 2020–2025 1 Harare: Ministry of Health and Child Care (unpublished), 2020; p. 1–48.

[12] Mangundu M, Roets L, Rensberg EJ Van, Africa S, Mangundu M. Accessibility of healthcare in rural Zimbabwe: The perspective of nurses and healthcare users. Afr J Prim Health Care Fam Med. 2020 May 14;12(1)1–7. 10.4102/phcfm.v12i1.2245 PMC7284155 32501024

[13] Dhlamini J. Strategy: An understanding of strategy for business and public policy settings. J Contemp Manage. 2022;19(2):108–134. 10.35683/jcm21073.161

[14] Brault I, Kilpatrick K, D’Amour D, et al. Role clarification processes for better integration of nurse practitioners into primary healthcare teams: A multiple-case study. Nurs Res Pract. 2014;2014:1–9. 10.1155/2014/170514 PMC4322308 25692039

[15] Verdejo C, Tapia-Benavente L, Schuller-Martínez B, Vergara-Merino L, Vargas-Peirano M, Silva-Dreyer AM. What you need to know about scoping reviews. Vol. 21. Medwave: Medwave Estudios Ltda; 2021. 10.5867/medwave.2021.02.8144 33914717

[16] Jung M, Han K. A concept analysis of gratitude in patients based on Rodgers. Evol Method Int J Cont. 2017;13(2):44–49. 10.5392/IJoC.2017.13.2.044

[17] Park JH, Lee EK. Nursing practice today. Nurs Pract Today [serial online]. 2021 [cited 2023 Nov 28];8(2):132–138. Available from: http://npt.tums.ac.ir/index.php/npt/article/view/132

[18] Ghahramanian A, Rassouli M, Zamanzadeh V, Valizadeh L, Asghari E. Good nursing care: Rodgers’ evolutionary concept analysis. Nurs Pract Today. 2020;7(1):12–20. 10.18502/npt.v7i1.2295

[19] Tricco AC, Lillie E, Zarin W, et al. PRISMA extension for scoping reviews (PRISMA-ScR): Checklist and explanation. Ann Intern Med. 2018;169(7):467–473. 10.7326/M18-0850 30178033

[20] Perry H, Crigler L, Hodgins S, Advisor T. Developing and strengthening community health worker programs at scale a reference guide and case studies for program managers and policymakers [homepage on the Internet]. 2014 [cited 2024 Jan 14]. Available from: https://chwcentral.org/wp-content/uploads/2018/02/pdf.usaid_.gov_pdf_docs_pa00jxwd.pdf

[21] Crigler L, Gergen J, Perry H. Supervision of community health workers. In: Perry H, Crigler L, editors. Developing and strengthening community health worker programs at scale: A reference guide and case studies for program managers and policy makers [homepage on the Internet]. [cited 2024 Jan 27]. Available from: https://coregroup.org/wp-content/uploads/2017/08/CHW-Reference-Guide-Chapter-10-Supervision-of-Community-Health-Workers.pdf

[22] Global Health Workforce Alliance. Integrating community health workers in national health workforce plans [homepage on the Internet]. Geneva: World Health Organisation. [cited 2022 Sep 11]. Available from: https://cdn.who.int/media/docs/default-source/health-workforce/ghwa_annual_report2014.pdf?sfvrsn=f00289de_3

[23] Kok MC, Ormel H, Broerse JEW, et al. Optimising the benefits of community health workers’ unique position between communities and the health sector: A comparative analysis of factors shaping relationships in four countries. Glob Public Health. 2017;12(11):1404–1432. 10.1080/17441692.2016.1174722 27133127

[24] Abdulraheem IS. Primary health care services in Nigeria: Critical issues and strategies for enhancing the use by the rural communities. J Public Health Epidemiol. 2012;4(1):5–13. 10.5897/JPHE11.133

[25] Colvin CJ. What motivates community health workers? Designing programs that incentivize community health worker performance and retention. In: Developing and strengthening community health worker programs at scale: A reference guide and case studies for program managers and policy makers [homepage on the Internet]. 2014 [cited 2023 Sep 17]. Available from: http://www.mchip.net/sites/default/files/mchipfiles/11_CHW_Incentives.pdf

[26] Aduo-Adjei K, Emmanuel O, Forster OM. The impact of motivation on the work performance of health workers (Korle Bu Teaching Hospital): Evidence from Ghana. Hosp Pract Res. 2016;1(2):45–50. 10.20286/hpr-010245

[27] Sarriot E, Davis T, Morrow M, Kabore T, Perry H. Motivation and performance of community health workers: Nothing new under the sun, and yet. Glob Health Sci Pract. 2021;9(4):716–724. 10.9745/GHSP-D-21-00627 34933969 PMC8691878

[28] Austin-Evelyn K, Rabkin M, MacHeka T, et al. Community health worker perspectives on a new primary health care initiative in the Eastern Cape of South Africa. PLoS One. 2017;12(3):e0173863. 10.1371/journal.pone.0173863 28301609 PMC5354377

[29] Krieger MGM, Wenham C, Nacif Pimenta D, et al. How do community health workers institutionalise: An analysis of Brazil’s CHW programme. Glob Public Health. 2022;17(8):1507–1524. 10.1080/17441692.2021.1940236 34161201

[30] Machado HSV, Melo EA, De Paula LGN. Family and community medicine in the supplementary health system in Brazil: Implications for the Unifed National Health System and for physicians. Vol. 35. Rio de Janeiro: Cadernos de Saude Publica. Fundacao Oswaldo Cruz; 2019. 10.1590/0102-311X00068419 31691777

[31] Minayo Gomez C, De Vasconcellos LCF, Machado JMH. A brief history of worker’s health in Brazil’s unified health system: Progress and challenges. Ciencia e Saude Coletiva. 2018;23(6):1963–1970. 10.1590/1413-81232018236.04922018 29972503

[32] Pinto RM, Da Silva SB, Soriano R. Community health workers in Brazil’s Unified Health System: A framework of their praxis and contributions to patient health behaviors. Soc Sci Med. 2012;74(6):940–947. 10.1016/j.socscimed.2011.12.025 22305469 PMC3299536

[33] Johnson CD, Noyes J, Haines A, et al. Learning from the Brazilian community health worker model in North Wales. Glob Health. 2013;9:25. 10.1186/1744-8603-9-25 PMC3681592 23764067

[34] Campbell JI, Eyal N, Musiimenta A, et al. Ugandan study participants experience electronic monitoring of antiretroviral therapy adherence as welcomed pressure to adhere. AIDS Behav. 2018;22(10):3363–3372. 10.1007/s10461-018-2200-8 29926301 PMC6309333

[35] Twimukye A, Naggirinya AB, Parkes-Ratanshi R, et al. Acceptability of a mobile phone support tool (Call for Life Uganda) for promoting adherence to antiretroviral therapy among young adults in a randomized controlled trial: Exploratory qualitative study. JMIR Mhealth Uhealth. 2021;9(6):e17418. 10.2196/17418 34121665 PMC8240800

[36] Ngabo F, Nguimfack J, Nwaigwe F, et al. Designing and implementing an innovative SMS-based alert system (RapidSMS-MCH) to monitor pregnancy and reduce maternal and child deaths in Rwanda [homepage on the Internet]. 2012 [cited 2024 Jan 27]. Available from: https://pmc.ncbi.nlm.nih.gov/articles/PMC3542808/pdf/PAMJ-13-31.pdf PMC3542808 23330022

[37] Crigler L, Hill K, Furth R, Bjerregaard D. Community health worker assessment and improvement matrix (CHW AIM): A toolkit for improving CHW programs and services. Bethesda, MD: University Research Co., LLC (URC), 2011 [cited 2022 Jul 31]; p. 1–146.

[38] Schleiff MJ, Aitken I, Alam MA, Damtew ZA, Perry HB. Community health workers at the dawn of a new era: 6. Recruitment, training, and continuing education. Health Res Policy Syst. 2021;19:113. 10.1186/s12961-021-00757-3 34641898 PMC8506097

[39] Wanduru P, Tetui M, Tuhebwe D, et al. The performance of community health workers in the management of multiple childhood infectious diseases in Lira, northern Uganda – A mixed methods cross-sectional study. Glob Health Action. 2016;9(1):33194. 10.3402/gha.v9.33194 27882866 PMC5122228

[40] Tetui M, Coe AB, Hurtig AK, et al. A participatory action research approach to strengthening health managers’ capacity at district level in Eastern Uganda. Health Res Policy Syst. 2017;15:110. 10.1186/s12961-017-0273-x 29297346 PMC5751402

[41] Carboni C, Maroto IJ, Galindo M, et al. Training-of-trainers program for community health workers involved in an innovative and community-based intervention against malaria among goldminers in the Guiana shield: A quality and effectiveness evaluation. Front Public Health. 2023;11:1306432. 10.3389/fpubh.2023.1306432 38259795 PMC10800722

[42] Vanden Bossche D, Willems S, Decat P. Understanding trustful relationships between community health workers and vulnerable citizens during the COVID-19 pandemic: A realist evaluation. Int J Environ Res Public Health. 2022;19(5):2496. 10.3390/ijerph19052496 35270193 PMC8909775

[43] Sripad P, McClair TL, Casseus A, Hossain S, Abuya T, Gottert A. Measuring client trust in community health workers: A multi-country validation study. J Glob Health. 2021;11:07009. 10.7189/jogh.11.07009 33763223 PMC7956104

[44] Kangovi S, Grande D, Trinh-Shevrin C. From rhetoric to reality – Community health workers in post-reform U.S. health care. N Engl J Med. 2015;372(24):2277–2279. 10.1056/NEJMp1502569 26061832 PMC4689134

[45] Kok MC, Vallières F, Tulloch O, et al. Does supportive supervision enhance community health worker motivation? A mixed-methods study in four African countries. Health Policy Plan. 2018;33(9):988–998. 10.1093/heapol/czy082 30247571 PMC6263021

[46] Kok MC, Vallières F, Tulloch O, et al. Does supportive supervision enhance community health worker motivation? A mixed-methods study in four African countries. Health Policy Plan. 2018;33(9):988–998. 10.1093/heapol/czy082 30247571 PMC6263021

[47] Findley S, Afenyadu G, Dalhat M, Gama E, Hafsat B, Mijinwaya S. Cost-effectiveness of alternative models of community health workers for promotion of maternal, newborn and child health in Northern Nigeria. Int J Transl Community Med. 2017;5(1):85–97. 10.19070/2333-8385-1700015

[48] Babiker A, El Husseini M, Al Nemri A, et al. Health care professional development: Working as a team to improve patient care [homepage on the Internet]. Vol. 14 2014 [cited 2024 January 14]. Available from: https://pmc.ncbi.nlm.nih.gov/articles/PMC4949805/pdf/sjp-14-9.pdf PMC4949805 27493399

[49] Mishra A. ‘Trust and teamwork matter’: Community health workers’ experiences in integrated service delivery in India. Glob Public Health. 2014;9(8):960–974. 10.1080/17441692.2014.934877 25025872 PMC4166967

[50] Tseng YH, Griffiths F, De Kadt J, et al. Integrating community health workers into the formal health system to improve performance: A qualitative study on the role of on-site supervision in the South African programme. BMJ Open. 2019;9(2). 10.1136/bmjopen-2018-022186 PMC6398712 30819698

[51] Baatiema L, Sumah AM, Tang PN, Ganle JK. Community health workers in Ghana: The need for greater policy attention. BMJ Glob Health. 2016 Dec 2; 1(4):e000141. 10.1136/bmjgh-2016-000141 PMC5321387 28588981

[52] Tuyisenge G, Crooks VA, Berry NS. Facilitating equitable community-level access to maternal health services: Exploring the experiences of Rwanda’s community health workers. Int J Equity Health. 2019 Nov 26;18(1):e022186. 10.1186/s12939-019-1065-4 PMC6880498 31771605

[53] Sarfraz M, Hamid S. Challenges in delivery of skilled maternal care – Experiences of community midwives in Pakistan. London: Springer; 2015. 10.1186/1471-2393-14-59 PMC3922011 24499344

[54] Amouzou A, Kozuki N, Gwatkin DR. Where is the gap?: The contribution of disparities within developing countries to global inequalities in under-five mortality. BMC Public Health. 2014;14(1):216. 10.1186/1471-2458-14-216 24581032 PMC3945794

[55] Olaniran A, Banke-Thomas A, Bar-Zeev S, Madaj B. Not knowing enough, not having enough, not feeling wanted: Challenges of community health workers providing maternal and newborn services in Africa and Asia. PLoS One. 2022;17:e0274110. 10.1371/journal.pone.0274110 36083978 PMC9462785

[56] Kulbok PA, Sgarlata L. Integrating community health workers into mainstream health systems through partnerships [homepage on the Internet]. Unpulished: Conference paper: 142nd APHA Annual Meeting and Exposition 2014. [cited 2023 Feb 11]. Available from: https://www.researchgate.net/publication/266785074

[57] Ploeg J, Wong ST, Hassani K, et al. Contextual factors influencing the implementation of innovations in community-based primary health care: The experience of 12 Canadian research teams. Prim Health Care Res Dev. 2019;20:e107. 10.1017/S1463423619000483 32800024 PMC8060818

[58] Mirzoev T, Das M, Ebenso B, et al. Contextual influences on the role of evidence in health policy development: What can we learn from six policies in India and Nigeria? Evid Policy. 2017;13(1):59–79. 10.1332/174426415X14454407579925

[59] Kwame A. Integrating traditional medicine and healing into the Ghanaian Mainstream health system: Voices from within. Qual Health Res. 2021;31(10):1847–1860. 10.1177/10497323211008849 33980093 PMC8446885

[60] Miiro C, Aoki Y, Shi S, Yakura H. Bridging the gap between community health workers’ digital health acceptance and actual usage in Uganda: Exploring key external factors based on technology acceptance model. Unpublished. 10.1371/journal.pdig.0001099 PMC12629443 41259341

[61] Ndima SD, Sidat M, Give C, Ormel H, Kok MC, Taegtmeyer M. Supervision of community health workers in Mozambique: A qualitative study of factors influencing motivation and programme implementation. Hum Resour Health. 2015;13(1):1–10. 10.1186/s12960-015-0063-x 26323970 PMC4556309

